# Can one long peritoneal dwell with icodextrin replace two short dwells with glucose?

**DOI:** 10.3389/fphys.2024.1339762

**Published:** 2024-07-10

**Authors:** Joanna Stachowska-Pietka, Jacek Waniewski, Anna Olszowska, Elvia Garcia-Lopez, Junfei Yan, Qiang Yao, Zofia Wankowicz, Bengt Lindholm

**Affiliations:** ^1^ Nalecz Institute of Biocybernetics and Biomedical Engineering, Polish Academy of Sciences, Warsaw, Poland; ^2^ Department of Clinical Science, Intervention and Technology, Division of Renal Medicine and Baxter Novum, Karolinska Institutet, Stockholm, Sweden; ^3^ Central Clinical Hospital of the Ministry of National Defence, Military Institute of Medicine, Warsaw, Poland; ^4^ Baxter Healthcare Corporation, Shanghai, China

**Keywords:** chronic kidney disease, peritoneal dialysis, icodextrin, ultrafiltration, sodium removal, glucose absorption, ultrafiltration efficiency

## Abstract

**Background:**

Due to the slower dissipation of the osmotic gradient, icodextrin-based solutions, compared to glucose-based solutions, can improve water removal. We investigated scenarios where one icodextrin-based long dwell (Extraneal) replaced two glucose-based exchanges.

**Methods:**

The three-pore model with icodextrin hydrolysis was used for numerical simulations of a single exchange to investigate the impact of different peritoneal dialysis schedules on fluid and solute removal in patients with different peritoneal solute transfer rates (PSTRs). We evaluated water removal (ultrafiltration, UF), absorbed mass of glucose (AbsGluc) and carbohydrates (AbsCHO, for glucose and glucose polymers), ultrafiltration efficiency (UFE = UF/AbsCHO) per exchange, and specified dwell time, and removed solute mass for sodium (ReNa), urea (ReU), and creatinine (ReCr) for a single peritoneal exchange with 7.5% icodextrin (Extraneal^®^) and glucose-based solutions (1.36% and 2.27%) and various dwell durations in patients with fast and average PSTRs.

**Results:**

Introducing 7.5% icodextrin for the long dwell to replace one of three or four glucose-based exchanges per day leads to increased fluid and solute removal and higher UF efficiency for studied transport groups. Replacing two glucose-based exchanges with one icodextrin exchange provides higher or similar water removal and higher daily sodium removal but slightly lower daily removal of urea and creatinine, irrespective of the transport type present in the case of reference prescription with three and four daily exchanges.

**Conclusion:**

One 7.5% icodextrin can replace two glucose solutions. Unlike glucose-based solutions, it resulted only in minor differences between PSTR groups in terms of water and solute removal with UFE remaining stable up to 16 h.

## 1 Introduction

The removal of excess water and uremic toxins is the main goal of peritoneal dialysis (PD). In conventional PD solutions, glucose at different concentrations is used as an osmotic agent to provide sufficient water removal (ultrafiltration, UF), together with solute removal. The higher the glucose concentration, the higher the osmotic gradient, which induces greater UF; however, glucose absorption increases. Moreover, the prolonged exposure to a higher glucose concentration might trigger local structural and functional changes within the peritoneal membrane that, in the long-term, may lead to insufficient water removal and, possibly, UF failure. As an alternative, to ameliorate these problems, an isosmotic solution (Extraneal^®^, Baxter) containing glucose polymers (7.5% icodextrin) instead of glucose as the primary osmotic agent can be used in longer dwells or in patients with insufficient UF to increase and prolong water removal due to the slower dissipation of the osmotic gradient created by icodextrin and to decrease the exposure to glucose.

The efficiency of water and solute removal is directly related to the characteristics of the peritoneal membrane and its transport capacity (permeability) that varies among patients and may change over time in long-term dialysis. Although recently more emphasis was given to fluid removal, the typical assessment of peritoneal membrane transport properties is based on small-solute transport and classification of individual patients according to their peritoneal small solute transfer rates (PSTRs) as fast, average, and slow transporters based on the peritoneal equilibration test (Morelle et al., 2021). This is partly due to the fact that, in the case of dwells with glucose-based solutions, a faster PSTR not only results in the faster and earlier equilibration of solutes but also the faster absorption of glucose and, consequently, lower UF, which, in turn, leads to the lower removal of urea and creatinine during the latter part of the dwell (Wang et al., 1998). This is in contrast to long icodextrin-based dwells, for which numerical simulations and also clinical trials suggest the lower dependence of UF on the PSTR and tendency to equalize water and sodium removal, especially among patients characterized by a fast and average PSTR (Lin et al., 2009; Akonur et al., 2016).

The current consensus is that small-solute removal should not be considered so strictly and treated as the only indicator of treatment adequacy, and that other factors should be also taken into account (Brown et al., 2020). Nowadays, modern PD prescriptions allow more possibilities to modify and optimize standard PD treatment regimens in order to not only provide effective treatment outcome in terms of fluid and solute removal but also to decrease glucose exposure that may harm the peritoneal membrane or reduce the number of exchanges as these changes may potentially protect residual renal function and increase the patient quality of life. The recent trend of the growing popularity of incremental dialysis is also consistent with the above fact (Blake et al., 2020). Incremental PD consists in the earlier initiation of PD with less frequent exchanges and/or with lower doses, with further modifications following changes occurring in membrane and renal function at the time of PD. It is very common to initiate PD therapy with only 2–3 exchanges per day, especially in Asia (Lo et al., 2001; Yan et al., 2022).

Various clinical studies suggest that icodextrin improved UF and lowered daily glucose absorption levels compared to glucose-based solutions (Goossen et al., 2020). In addition, the differences in mechanisms involved in the water removal for icodextrin and glucose-based solutions were investigated experimentally and using mathematical modeling (Morelle et al., 2018; Morelle et al., 2023); however, the magnitude of its superiority requires further research. In this study, we applied the three-pore model to simulate various PD schedules and their impact on fluid and solute removal under well-specified and controlled conditions to investigate whether glucose-based exchanges can be replaced with 7.5% icodextrin in the same patients and, if yes, to which extent? We especially address one question: can one icodextrin-based long dwell replace two exchanges with glucose-based solutions? Since the problem of insufficient water removal is mainly observed in patients with a fast PSTR, we primarily focus on this group, but we also verify to which extent answers to this question are transferable to patients with other transport status.

## 2 Materials and methods

### 2.1 Model overview

All simulations presented in this study are based on the extended version of the three-pore model for peritoneal transport (including dwells with 7.5% icodextrin) described in detail below. The proposed model is based on the classical three-pore approach applied to describe the peritoneal transport of water and solutes. In addition, icodextrin hydrolysis and its impact on peritoneal transport are taken into account; in the present study, we used a minimal model, i.e., a simplified version of the models of icodextrin hydrolysis by α-amylase proposed by Akonur et al. (2015) and Stachowska-Pietka et al. (2023), which is still sufficient to provide an accurate description of clinical data.

The applied model describes changes in the intraperitoneal volume and solute concentrations in the dialysate. The following solutes are considered in the model: small solutes (urea, creatinine, glucose, and sodium) and icodextrin polysaccharides [aggregated into seven fractions, i.e., glucose polymer-size classes, with molecular weight cut-off values up to 1.08 kDa (fraction 1), 4.44 kDa (fraction 2), 9.89 kDa (fraction 3), 21.4 kDa (fraction 4), 43.5 kDa (fraction 5), 66.7 kDa (fraction 6), and over 66.7 kDa (fraction 7), as described in Stachowska-Pietka et al. (2023)].

### 2.2 Model-based simulations

Numerical simulations of peritoneal transport during a single exchange were carried out for a typical patient with an average PSTR according to PET. The characteristics of a “typical peritoneal membrane” were taken based on the adjustment of the model to the clinical data of nine patients undergoing PD with glucose 2.27% solution, described previously in Heimburger et al. (1992). More precisely, the fractional small-pore UF coefficient (α_SP_) and unrestricted pore area over the (unit) diffusion path length distance (A_0_/Δx) were adjusted to clinical data from Heimburger et al. (1992), and peritoneal fluid absorption of 1.2 mL/min was taken into account based on the volume marker absorption from the peritoneal cavity. For each solute, the diffusive permeability of the peritoneal membrane (PS) was calculated according to the standard formulas from the three-pore approach, as proposed and applied previously based on the adjusted value of A_0_/Δx (Rippe and Levin, 2000; Rippe et al., 2004; Waniewski, 2013). The diffusive permeability of urea was adjusted separately to A_0_/Δx (with a slightly higher value than the theoretical one) to obtain a precise description of urea removal during the peritoneal exchange. This remains in agreement with clinical observations that higher values of diffusive transport parameters for urea were observed during PD exchanges related to its transport across the peritoneal tissue cells. The model describes data with high accuracy with an average relative error per measurement point of 0.01, as presented in Supplementary Figure S1.

The obtained characteristics of the peritoneal membrane were taken to provide the simulation of glucose and icodextrin exchanges. The typical values of the peritoneal fluid absorption rate measured using a volume marker range from 0.7 up to 1.6 mL/min but typically remain below 1 mL/min (Waniewski, 2013). The mean value of peritoneal absorption measured in Heimburger et al. (1992) was 1.2 ± 0.6 mL/min, whereas a lower mean value of 0.67 ± 0.39 mL/min was found in Stachowska-Pietka et al. (2023). Therefore, to provide reliable simulations for a typical patient, we considered an average value of those two, 0.9 mL/min. The typical parameters for icodextrin hydrolysis by amylase were taken based on the average values obtained using the minimal model for the analysis of clinical data on long-term icodextrin-exposed patients undergoing 16-h 7.5% icodextrin dwells, published previously by Olszowska et al. (2019). The simulation of a single exchange of 2 L dialysis fluid with 2.27% and 1.36% glucose lasting up to 8 h and with 7.5% icodextrin lasting up to 16 h was performed with parameters as presented in Supplementary Table S1.

The simulation of a single exchange of 2 L dialysis fluid with 2.27% and 1.36% glucose lasting up to 8 h and with 7.5% icodextrin lasting up to 16 h was performed with parameters as presented in Supplementary Table S1. The initial concentrations of the solutes in the dialysate were calculated taking into account the dilution of solute concentrations in fresh dialysis fluid (according to specifications by the manufacturer) by the residual peritoneal volume. Numerical simulations for a patient with a fast PSTR were performed with the rescaled solute diffusive permeabilities of the peritoneal membrane (PSs), following the changes in A_0_/Δx (increased by a factor of 1.6), as previously proposed by Oberg (2021).

### 2.3 Minimal three-pore model for icodextrin transport

In order to simulate peritoneal dwells with icodextrin, the classical three-pore model was extended. Since the modeling of hydrolysis was out of scope of this study, we applied a model that would describe the kinetics of icodextrin fractions without a detailed description of its hydrolysis. We call this model a minimal model as it is a simplified version of the models that take into account icodextrin hydrolysis by α-amylase proposed by Akonur et al. (2015) and Stachowska-Pietka et al. (2023), which is still sufficient to provide an accurate description of clinical data.

A simplified kinetics was proposed using pseudo-first-order degradation kinetics to describe the net decrease in icodextrin fraction mass in the dialysate (fractions 2–7) that was related to the hydrolysis with a constant degradation rate $k_{i}$ (Akonur et al., 2015; Stachowska-Pietka et al., 2023). Therefore, in addition to peritoneal kinetics, a decrease in the mass of fractions 2–7 corresponding to the hydrolysis can be calculated as $k_{i}\cdot C_{D,amylase}\cdot \left(V_{D}\cdot C_{D,Ico_{i}}\right)$ , where $V_{D}\cdot C_{D,Ico_{i}}$ denotes the mass of fraction *i*. Similar to the previous approaches, we assume additionally that the degradation rate depends on the amylase concentration in dialysate $C_{D,amylase}$ . The (net) mass of icodextrin polymers from fractions 2 to 7 degraded by the amylase activity (expressed in moles/min) would increase the molar mass of fraction 1 (by $2\sum_{i=2,...,7}k_{i}\cdot C_{D,amylase}\cdot \left(V_{D}\cdot C_{D,Ico_{i}}\right)$ ) additionally to its increase related to the degradation of polymers present already in fraction 1 ( $k_{1}\cdot C_{D,amylase}\cdot \left(V_{D}\cdot C_{D,Ico_{1}}\right)$ .

Similar to the classical three-pore model, the minimal model was adjusted to the clinical data on long-term icodextrin-exposed patients undergoing 16-h 7.5% icodextrin dwells, published previously by Olszowska et al. (2019). The individual kinetics of the intraperitoneal volume and the concentration of glucose, urea, creatinine, and icodextrin fractions 1–7 were taken into account. The goodness of the minimal model to predict peritoneal transport in icodextrin dwells was high with an average relative error per measurement point of 0.032 (Supplementary Figures S2, S3).

### 2.4 Calculation of treatment efficiency

For each session, the removal of urea, creatinine, and sodium (ReU, ReCr, and ReNa, respectively) was calculated as the difference between the mass of the solute removed in the spent dialysate and the solute mass instilled. The absorption of glucose from glucose-based solutions and carbohydrates (polysaccharides) from icodextrin-based solution during the peritoneal exchange was calculated as the difference between the solute mass infused in the fresh solution and its mass removed in the drained fluid. The total absorption of carbohydrates, AbsCHO, was calculated as the total mass of glucose absorbed (in the case of glucose-based solution) or the sum of the total glucose and carbohydrate mass absorbed (for 7.5% icodextrin) during a single session or for a whole schedule. In addition, the total mass of glucose absorbed, AbsGluc, was calculated for each schedule.

The removal of fluid was evaluated based on the UF and calculated as the difference between the final volume decreased by the residual volume and the infused volume. The efficiency of dialysis solution to remove water, i.e., ultrafiltration efficiency UFE, was defined as UF divided by the total absorption of carbohydrates, AbsCHO, during a specified time—a single exchange with various dwell times or daily.

### 2.5 Numerical simulations

The parameters for the classical and minimal three-pore model were estimated using the “lsqnonlin” function implemented with the trust–region–reflective algorithm implemented to minimize the sum of squared differences between the modeled and measured values for the intraperitoneal volume and concentration of glucose, urea, creatinine, and icodextrin fractions (for the minimal model only) in the dialysate.

The numerical simulation of glucose and icodextrin-based dwells was performed using the minimal three-pore model that was implemented and solved using the commercial software package MATLAB with the built-in function “ode45,” which is based on an explicit Runge–Kutta formula with variable step size. Computer-based simulations were performed according to the concept presented in Sections 2.1–2.4 and with the parameters presented in Supplementary Table S1.

## 3 Results

**FIGURE 1:**
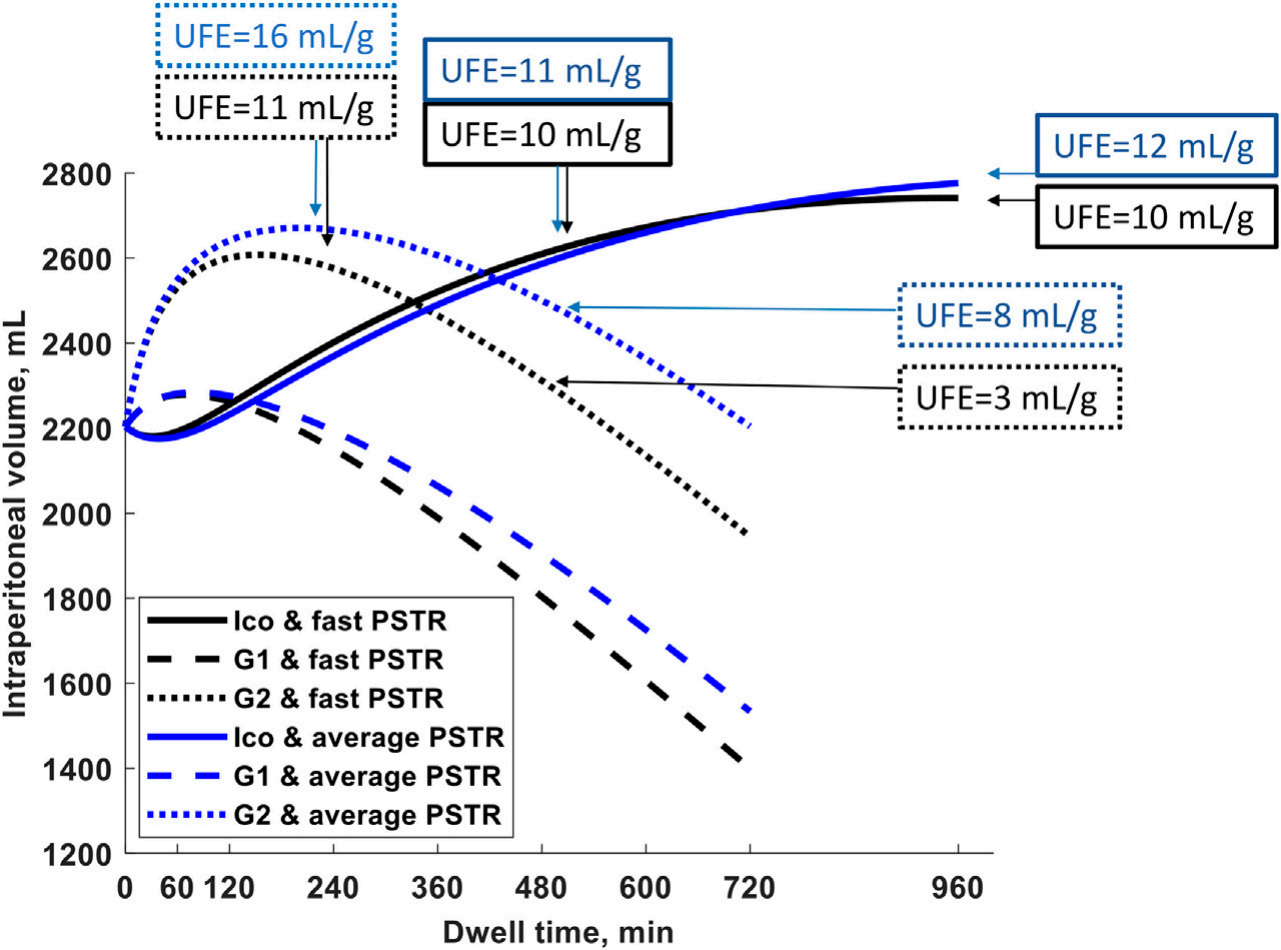
Intraperitoneal volume as a function of dwell time during a 12-h exchange with 1.36% (G1) and 2.27% glucose (G2) and during a 16-h exchange with Extraneal (Ico, 7.5% icodextrin-based solution) for a patient with fast peritoneal solutes transfer rate (PSTR) and average PSTR. The figure shows marked differences between the three solutions in the intraperitoneal volume with transitory increases in the two glucose-based solutions that are accentuated by a faster PSTR, while the steady increase of volume up to 14–16 h with 7.5% icodextrin (Extraneal) is not influenced much by the PSTR. The ultrafiltration efficiency, UFE (volume of water removed in mL per gram absorbed carbohydrates), for G2 and 7.5% icodextrin (Extraneal) and for both PSTR groups is noted at specified dwell times (4 and 8 h for G2 and 8 and 16 h for Ico), showing a decrease in UFE in glucose-based solutions already after the 4-h dwell, whereas UFE remains stable even up to 16 h in the icodextrin-based solution.

The changes in the intraperitoneal volume during a single exchange with 2.27% and 1.36% glucose and icodextrin-based (Extraneal) solutions are presented in Figure 1 for patients with an average and fast PSTR. In the case of shorter exchanges, glucose-based solutions are more efficient in water removal than 7.5% icodextrin, whereas icodextrin provides greater UF for longer dwells (Figure 1). Initially, for glucose-based solutions, the intraperitoneal volume increases. However, the dominance of peritoneal fluid absorption over ultrafiltration during the later phase of the peritoneal dwell results in the thereafter observed steady decrease in the intraperitoneal volume and, thus, the appearance of negative ultrafiltration for long-enough dwells (Figure 1). The appearance of negative ultrafiltration, corresponding to the absorption of fluid, can be observed earlier in the case of solutions with a lower glucose concentration, as well as in patients with faster transport status, due to the faster dissipation of the osmotic gradient (Table 1). This is in contrast to the icodextrin-based exchanges for which the intraperitoneal volume gradually increases up to 14–16 h of the peritoneal dwell (Figure 1; Table 1). However, the model predicts that for long-enough dwells with 7.5% icodextrin (exceeding 14–16 h), the fluid absorption rate will finally exceed the UF rate, eventually leading to a slow decrease in the intraperitoneal volume (instead of the observed increase) that would occur sooner in the case of patients with a faster PSTR. In contrast to glucose-based solutions, the changes in the intraperitoneal volume during dwells with 7.5% icodextrin are similar among patients from different transfer groups (difference in UF for a single exchange is smaller than 65 mL) (Figure 1; Table 1).

**TABLE 1:** Simulated ultrafiltration (UF), absorbed carbohydrates (AbsCHO for glucose and glucose polymers), ultrafiltration efficiency (UFE = UF/AbsCHO) per single exchange and specified dwell time, and removed solute mass calculated for sodium (ReNa), urea (ReU), and creatinine (ReCr) for a single peritoneal exchange with icodextrin-based (Extraneal) and glucose-based solutions (2.27% and 1.36%) and different dwell durations for a patient with a fast and average peritoneal solute transfer rate (PSTR). NA, not applicable due to net fluid absorption.

Solution	Icodextrin			Glucose 2.27%					Glucose 1.36%				
Dwell duration	10 h	12 h	16 h	4 h	6 h	8 h	10 h	12 h	4 h	6 h	8 h	10 h	12 h
Transfer group	Fast PSTR												
UF, mL	469	510	538	373	262	109	−68	−258	−53	−214	−399	−598	−804
AbsCHO, g	45.2	50.1	54.8	34.0	37.8	39.9	41.2	42.0	20.8	22.8	23.9	24.5	25.0
UFE, mL/g	10.4	10.2	9.8	11.0	6.9	2.7	NA	NA	NA	NA	NA	NA	NA
ReNa, mmoL	75.1	80.9	85.2	51.1	42.2	24.5	1.9	−23.5	0.1	−19.2	−43.4	−70.2	−98.5
ReU, g	2.65	2.70	2.74	2.5	2.4	2.3	2.1	1.9	2.1	1.9	1.7	1.5	1.3
ReCr, g	0.21	0.21	0.22	0.17	0.18	0.18	0.16	0.15	0.15	0.15	0.14	0.12	0.10

Transfer group	Average PSTR												
UF, mL	457	512	573	463	403	297	160	1	−10	−140	−299	−478	−669
AbsCHO, g	39.7	43.9	47.9	28.3	32.8	36.0	38.3	39.9	18.1	20.7	22.4	23.6	24.4
UFE, mL/g	11.5	11.7	12.0	16.4	12.3	8.2	4.2	0.0	NA	NA	NA	NA	NA
ReNa, mmoL	73.4	80.8	89.3	51.7	51.4	42.6	27.9	8.7	0.9	−13.1	−32.3	−55.2	−80.7
ReU, g	2.6	2.7	2.8	2.3	2.4	2.4	2.3	2.2	2.0	1.9	1.8	1.7	1.4
ReCr, g	0.20	0.21	0.22	0.15	0.17	0.17	0.17	0.16	0.13	0.14	0.14	0.13	0.11

During peritoneal dwells, the concentration of the osmotic agents in dialysate, glucose and icodextrin, slowly decreases. The absorption of glucose occurs during the whole dwell period with glucose-based solutions, with 42 g and 25 g of glucose being absorbed after 12 h for a patient with a fast PSTR and 2.27% and 1.36% glucose, respectively, as presented in Table 1. In the case of icodextrin-based solutions, the amount of carbohydrates absorbed during the peritoneal dwell, AbsCHO, is mainly related to the absorption of glucose polymers composing icodextrin, while glucose is removed from the body in the dialysis fluid. After a 16-h dwell, the absorption of carbohydrates reaches net AbsCHO = 55 g for fast transporters (including an estimated amount of 5 g of glucose removed from blood to the dialysate) and AbsCHO = 48 g for patients with an average PSTR (Table 1). Moreover, the efficiency of icodextrin to remove water, UFE, remains relatively stable with values close to 10 mL/g for a fast PSTR and 11–12 mL/g for average transporters. Unlike for 7.5% icodextrin, the efficiency of glucose-based solutions, UFE, does not remain stable but decreases with the dwell length, being higher in the case of dialysis fluid with higher tonicity (Table 1).

Sodium removal follows water transport, being the highest in the case of dwells with 7.5% icodextrin for dwells longer than 6 h in patients with a fast and average PSTR, Table 1. Moreover, in the case of the usage of icodextrin-based solutions, sodium removal increases with dwell time, while the removal of sodium in dwells with glucose-based solutions increases initially but then decreases after 4–6 h of peritoneal dwells due to the decrease in the peritoneal dialysate volume (Table 1). Consequently, for long-enough dwells, effective absorption of sodium among fast transporters is predicted by the model to occur after 12 h and 6 h for 2.27% and 1.36% glucose, respectively, as indicated by negative values of ReNa in Table 1. In patients with a fast PSTR, the removal of urea and creatinine already decreases after 4 h of glucose dwells (Table 1). In the case of patients with an average PSTR, the corresponding decrease in the urea and creatinine mass removed is observed later, after 6–8 h (Table 1). This is in contrast to 7.5% icodextrin, for which a slight increase in the urea and creatinine mass removed is observed throughout the dwell time and for patients with all transfer types (Table 1). Furthermore, when comparing the removal of small solutes for dwells with the same duration, the removal of solutes is higher with 2.27% glucose than with 7.5% icodextrin in the case of shorter dwells, while for longer dwells, superiority of icodextrin-based solution in terms of the solute mass removed is observed for patients with all types of peritoneal transfer (Table 1) (not shown for slow transporters).

Let us consider a patient with a fast PSTR undergoing initially three 8-h exchanges per day with 2 L of 1.36% or 2.27% glucose-based solution (presented as reference prescriptions in Table 2) and compare it with new scenarios in which one glucose-based exchange (A_1_ and C_1_, and B_1_ with an additional change in glucose concentration in one exchange) or two glucose dwells (scenarios D_1_ and E_1_) were replaced by one exchange of 7.5% icodextrin. In scenarios A_1_–C_1_, a single 12-h exchange with the icodextrin-based solution is assumed, followed by two 6-h exchanges with 1.36% or 2.27% glucose-based solutions, whereas in scenarios D_1_ and E_1_, two glucose 8-h exchanges are replaced by one 16-h dwell with 7.5% icodextrin (Table 2). Based on the results presented in Table 1, the corresponding fluid and solute removals for all theoretical schedules were calculated and presented in Table 2. As expected, the reference prescription with 2.27% glucose provides the higher removal of fluid and solutes with daily UFE = 2.7 mL/g if compared to 1.36% glucose, for which the net absorption of fluid and sodium is predicted (negative UF and ReNa) (Table 2). However, the higher water removal for 2.27% glucose is accompanied by higher glucose absorption (>48 g daily) (Table 2). The substitution of one glucose dwell (from the reference prescription) by one icodextrin dwell leads to the increased removal of water and solutes, even in the case of shorter dwells of glucose solutions, as well as higher daily UFE (scenarios A_1_ and C_1_; Table 2). Moreover, although the total absorption of carbohydrates increases, the daily absorption of glucose for scenarios A_1_ and C_1_ is lower than in all reference prescriptions, i.e., 40.8 g and 70.9 g for scenarios A_1_ and C_1_, respectively. Furthermore, in the case of reference prescription with 2.27% glucose, the fluid and solute removal is also higher when replacing one glucose exchange by one exchange with icodextrin, together with a lowering glucose concentration in one of the two remaining glucose-based exchanges, scenario B_1_ (Table 2). Similarly, in the case of a patient with an average PSTR, the removal of water and solutes is also higher for scenarios A_1_ and C_1_, as can be calculated from the results presented in Table 1.

**TABLE 2:** Comparison of reference prescription of three exchanges/day with 2L of glucose 1.36% (G1) or 2.27% (G2) solution with new scenarios (A_1_–E_1_), where one icodextrin-based exchange replaces one or two glucose-based exchanges, in terms of fluid and solute removal^a^ for patients with a fast peritoneal transfer rate (PSTR).

Reference prescription Fast PSTR				New scenarios					
				A_1_	B_1_	C_1_	D_1_	E_1_	
No. of dwells	Three exchanges/day			Three exchanges/day			Two exchanges/day		
Dwell time^b^	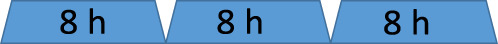			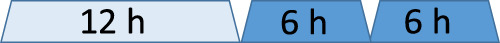			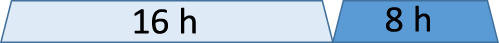		
Dialysis fluid	3xG1	3xG2		1xIco+2xG1	1xIco+1xG1+1xG2	1xIco+2xG2	1xIco+1xG1	1xIco+1xG2	
UF, mL	−1,198	326		82	558	1,034	139	647	
AbsGluc, g	71.6	119.8		40.8	55.9	70.9	19.1	35.1	
AbsCHO, g	71.6	119.8		95.6	110.7	125.7	78.6	94.7	
UFE, mL/g	N.A.	2.7		0.9	5.0	8.2	1.8	6.8	
ReNa, mmol	−130.2	73.6		42.5	103.9	165.3	41.8	109.7	
ReU, g	5.2	6.9		6.6	7.1	7.6	4.5	5.0	
ReCr, g	0.41	0.53		0.51	0.54	0.58	0.35	0.39	

^a^ Ultrafiltration (UF), absorbed glucose (AbsGluc), and carbohydrates (AbsCHO for glucose and glucose polymers), ultrafiltration efficiency (UFE), and removed solute mass for sodium (ReNa), urea (ReU), and creatinine (ReCr) were calculated for the whole schedule.

^b^ Glucose-based exchanges are denoted by dark blue, and icodextrin-based exchanges by light blue.

The substitution of two glucose-based exchanges from the reference prescription with 1.36% and 2.27% glucose by one 16-h icodextrin exchange leads to an increase in UF from −1,198 and 326 mL to 139 (+1,337 mL) and 647 (+321 mL) mL, respectively (scenarios D_1_ and E_1_; Table 2). This change also leads to improved sodium removal (increase of ReNa by 172 and 36 mmol for 1.36% and 2.27% glucose, respectively) and higher UFE (Table 2). The observed increase in AbsCHO for scenario D_1_ and decrease for E_1_ correspond to the increased total absorption of carbohydrates, whereas glucose absorption per day is lower (only 19 g and 35 g, respectively, for scenarios D_1_ and E_1_). However, the corresponding removal of urea and creatinine decreases in scenarios D_1_ and E_1_ by 13% and 15%, respectively, for 1.36% glucose solution and by 28% and 26% for 2.27% glucose solution (Table 2). Similarly, when substituting two glucose exchanges by one long dwell with the icodextrin-based solution in a patient with an average PSTR, this results in higher UF and higher UFE and sodium removal but not higher urea and creatinine removal.

Table 3 shows water and solute removal for a patient with a fast PSTR undergoing four daily exchanges as reference prescription, three short 4-h exchanges and one long 12-h exchange each with 2 L of solution. We consider different scenarios—A_2_ and D_2_—replacing one glucose exchange from initial reference scenarios by one icodextrin dwell, and scenarios B_2_ and C_2_ additionally with a different mix use of 1.36% and 2.27% exchanges (Table 3). In general, 7.5% icodextrin is more effective in fluid and solute removal not only if one long glucose dwell is replaced by one icodextrin dwell, scenarios A_2_ and D_2_, but also if additionally, the concentration in one glucose exchange is decreased (scenario C_2_ in Table 3). However, a further decrease in glucose concentration, as in scenario B_2_, although resulting in similar or slightly higher removal of solutes and higher UFE, leads to lower UF than reference prescription with glucose 2.27% (Table 3).

**TABLE 3:** Comparison of the reference prescription of four exchanges/day with 2L of glucose 1.36% (G1) or 2.27% (G2) solution with new scenarios (A_2_–F_2_), where one icodextrin-based exchange replaces one or two glucose-based exchanges, in terms of fluid and solute removal^a^ for patients with a fast peritoneal transfer rate (PSTR).

Reference prescription Fast PSTR			New scenarios					
			A_2_	B_2_	C_2_	D_2_	E_2_	F_2_
No. of dwells	Four exchanges/day		Four exchanges/day				Three exchanges/day	
Dwell time^b^	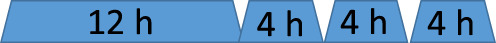		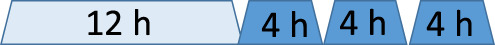				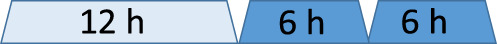	
Dialysis fluid	4xG1	4xG2	1xIco+3xG1	1xIco+2xG1+1xG2	1xIco+1xG1+2xG2	1xIco+3xG2	1xIco+2xG1	1xIco+2xG2
UF, mL	−963	860	351	777	1,203	1,628	82	1,034
AbsGluc, g	87.3	143.9	57.6	70.8	84.0	97.2	40.8	70.9
AbsCHO, g	87.3	143.9	112.4	125.6	138.8	152.0	95.6	125.7
UFE, mL/g	N.A.	6.0	3.1	6.2	8.7	10.7	0.9	8.2
ReNa, mmol	−98.1	129.9	81.3	132.3	183.3	234.3	42.5	165.3
ReU, g	7.5	9.3	8.9	9.3	9.7	10.1	6.6	7.6
ReCr, g	0.55	0.67	0.66	0.68	0.71	0.73	0.51	0.58

^a^ Ultrafiltration (UF), absorbed glucose (AbsGluc), and carbohydrates (AbsCHO for glucose and glucose polymers), ultrafiltration efficiency (UFE), and removed solute mass for sodium (ReNa), urea (ReU), and creatinine (ReCr) were calculated for the whole schedule.

^b^ Glucose-based exchanges are denoted by dark blue, and icodextrin-based exchanges, by light blue.

The replacement of two glucose exchanges by one icodextrin exchange, without changing the glucose concentration in the remaining exchanges, leads to an increase in water and sodium removal, which is higher for 2.27% than for 1.36% solutions, scenarios E_2_ and F_2_ (Table 3). It also results in lower glucose uptake (41 g and 71 g for scenarios E_2_ and F_2_, respectively, although AbsCHO is lower only in the case of 2.27% glucose) and higher UFE (Table 3). However, such a replacement also leads to the lower removal of urea and creatinine by 12% and 7%, respectively, for 1.36% glucose (scenario E_2_) and by 18% and 15%, respectively, for 2.27% glucose (scenario F_2_) (Table 3). A further increase in the effectiveness of water removal can be obtained by prolonging icodextrin exchange and shortening glucose dwells, for example, from 12 h + 2 × 6 h to 16 h + 2 × 4 h (UFE equal to 4.5 and 10.5 mL/g for 1.36% and 2.27% glucose, respectively, calculated based on Table 1). On the other hand, although scenario E_2_ is more efficient in water and sodium removal than reference prescription with initial exchanges with 2.27% glucose (Table 3), an additional decrease in the glucose concentration in one of the remaining exchanges (scenario B_1_ in Table 2) leads to lower water and sodium removal. Similarly, a beneficial effect of icodextrin in water and sodium removal (in case of scenarios E_2_ and F_2_) is also present if considering patients with an average PSTR.

Detailed results for patients with an average PSTR having 3–4 exchanges per day have a similar trend and are presented in Tables 4, 5.

**TABLE 4:** Comparison of the reference prescription of three exchanges/day with 2L of glucose 1.36% (G1) or 2.27% (G2) solution with new scenarios (A_1_–E_1_), where one icodextrin-based exchange replaces one or two glucose-based exchanges, in terms of fluid and solute removal^a^ for patients with an average peritoneal transfer rate (PSTR).

Reference prescription Average PSTR			New scenarios				
			A_1_	B_1_	C_1_	D_1_	E_1_
No. of dwells	Three exchanges/day		Three exchanges/day			Two exchanges/day	
Dwell time^b^	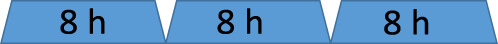		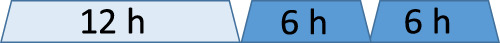			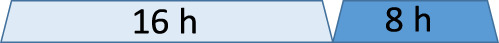	
Dialysis fluid	3xG1	3xG2	1xIco+2xG1	1xIco+1xG1+1xG2	1xIco+2xG2	1xIco+1xG1	1xIco+1xG2
UF, mL	−897	891	232	775	1,318	274	870
AbsGluc, g	67.2	108.1	36.8	49.0	61.2	17.6	31.3
AbsCHO, g	67.2	108.1	85.2	97.4	109.5	70.3	83.9
UFE, mL/g	N.A.	8.2	2.7	8.0	12.0	3.9	10.4
ReNa, mmol	−96.9	127.9	54.6	119.1	183.5	57.0	132.0
ReU, g	5.5	7.3	6.6	7.1	7.6	4.6	5.2
ReCr, g	0.41	0.52	0.48	0.51	0.54	0.35	0.39

^a^ Ultrafiltration (UF), absorbed glucose (AbsGluc), and carbohydrates (AbsCHO for glucose and glucose polymers), ultrafiltration efficiency (UFE), and removed solute mass for sodium (ReNa), urea (ReU), and creatinine (ReCr) were calculated for the whole schedule.

^b^ Glucose-based exchanges are denoted by dark blue, and icodextrin-based exchanges, by light blue.

**TABLE 5:** Comparison of the reference prescription of four exchanges/day with 2L of glucose 1.36% (G1) or 2.27% (G2) solution with new scenarios (A_2_–F_2_), where one icodextrin-based exchange replaces one or two glucose-based exchanges, in terms of fluid and solute removal^a^ for patients with an average peritoneal transfer rate (PSTR).

Reference prescription Average PSTR			New scenarios					
			A_2_	B_2_	C_2_	D_2_	E_2_	F_2_
No. of dwells	Four exchanges/day		Four exchanges/day				Three exchanges/day	
Dwell time^b^	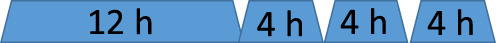		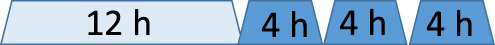				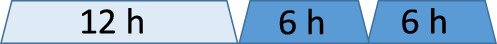	
Dialysis fluid	4xG1	4xG2	1xIco+3xG1	1xIco+2xG1+1xG2	1xIco+1xG1+2xG2	1xIco+3xG2	1xIco+2xG1	1xIco+2xG2
UF, mL	−699	1,390	483	955	1,428	1901	232	1,318
AbsGluc, g	78.6	124.7	49.7	59.9	70.1	80.3	36.8	61.2
AbsCHO, g	78.6	124.7	98.1	108.3	118.5	128.7	85.2	109.5
UFE, mL/g	N.A.	11.1	4.9	8.8	12.1	14.8	2.7	12.0
ReNa, mmol	−77.9	163.8	83.6	134.3	185.1	235.8	54.6	183.5
ReU, g	7.3	9.1	8.5	8.9	9.3	9.6	6.6	7.6
ReCr, g	0.50	0.61	0.59	0.61	0.63	0.65	0.48	0.54

^a^ Ultrafiltration (UF), absorbed glucose (AbsGluc), and carbohydrates (AbsCHO for glucose and glucose polymers), ultrafiltration efficiency (UFE), and removed solute mass for sodium (ReNa), urea (ReU), and creatinine (ReCr) were calculated for the whole schedule.

^b^ Glucose-based exchanges are denoted by dark blue, and icodextrin-based exchanges, by light blue.

## 4 Discussion

This study shows that in PD patients who receive 3–4 daily exchanges with 1.36% or 2.27% glucose-based solutions, one icodextrin-based long dwell can replace two exchanges with glucose-based solutions in terms of the removal of fluid and sodium. Such a change in the prescription was found to provide higher or similar removal of water and sodium but slightly lower daily removal of urea and creatinine, independent of the PSTR. Consequently, there could be potential positive clinical implications linked to the better control of fluid status and improved quality of life because of less intrusion in regular life due to reduced number of exchanges. These potential advantages should be weighed against the potential disadvantage of the lower removal of urea and creatinine and other uremic toxins.

The introduction of icodextrin into reference prescriptions comprising 3–4 exchanges per day of glucose-based solutions leads to substantial increase in fluid and solute removal, lower glucose absorption, and higher UF efficiency for patients from all transport groups. The observed higher values of AbsCHO in the new prescriptions with icodextrin (although glucose absorption was lower) were related not only to the larger infused mass of CHO but also dwell duration. In fact, AbsCHO for icodextrin predicted for the same dwell duration and irrespective of transport groups was similar to that for 2.27% glucose being 39–44 g/8-h and 36–40 g/8-h dwell, respectively, and similar to the values of AbsCHO found in the MIDAS study—38 g/8-h and 39 g/8-h dwell, respectively (Mistry et al., 1994). Moreover, in contrast to dwells with the glucose-based solution, the absorption of icodextrin metabolites does not result in the observed hyperglycemia and hyperinsulinemia (Gokal et al., 2002). This is mainly due to the fact that during peritoneal dwell, icodextrin is hydrolyzed by α-amylase to maltose and larger glucose polymers, and further metabolism to glucose by maltase occurs rather intracellularly (Mistry et al., 1994; Gokal et al., 2002). The results for other treatment schedules, i.e., with different numbers and duration of dwells, can be easily calculated based on the results given in Table 1. Our predictions of the effects of the usage of icodextrin vs. glucose-based solutions in terms of fluid and solute (sodium, urea, and creatinine) removal agree with the clinical observations in randomized control studies in different groups of patients with the same dialysis dosage (Lin et al., 2009; Qi et al., 2011; Goossen et al., 2020). Similar results were previously predicted by numerical simulations of automated PD (APD) and continuous ambulatory PD (CAPD) with glucose and icodextrin solutions (Akonur et al., 2016). The tendency of icodextrin to equalize water and sodium removal among patients with different PSTRs is in contrast to the glucose-based solutions in which the lower removal of water, urea, and creatinine is observed in patients with a fast PSTR and longer dwells (Wang et al., 1998). Moreover, a study looking within the same group of patients at the effect on fluid and solute removal of a night exchange of icodextrin vs. a night exchange of 1.36%, 2.27%, or 3.86% glucose demonstrated that UF and weekly removal of creatinine and urea were superior with icodextrin (Paniagua et al., 2012). Another advantage of using icodextrin is the reduction in the metabolic burden of glucose overexposure, which may be especially significant in the management of fast transport diabetic patients on PD by facilitating metabolic control (Paniagua et al., 2009).

Although we did not specifically analyze consequences for PD patients undergoing APD, our results are also relevant for APD patients using the icodextrin-based solution for the long daytime dwell (Plum et al., 2002). Interestingly, a study showed that a CAPD regimen with one icodextrin-containing and two glucose-containing solutions may contribute to a better preservation of residual renal function and a more biocompatible regimen, with similar dialysis adequacy to a regimen of four daily glucose-containing exchanges (Yoon et al., 2014). This is well-aligned with the study result in our math model. Another recent application is to use one 7.5% icodextrin (Extraneal) solution to replace two glucose-containing solutions as part of the prescription for incremental PD (Fernandes et al., 2023). By using the modified three-pore model, Guest et al. evaluated incremental CAPD prescriptions with 1–3 dwells/day using 2 L of icodextrin and glucose-based solutions and found that two dwells/day (including one Extraneal and one glucose solution) in patients with a glomerular filtration rate of at least 6 mL/min per 1.73 m^2^ are sufficient to achieve clearance goals in all four types of peritoneal transport (Guest et al., 2017).

**FIGURE 2:**
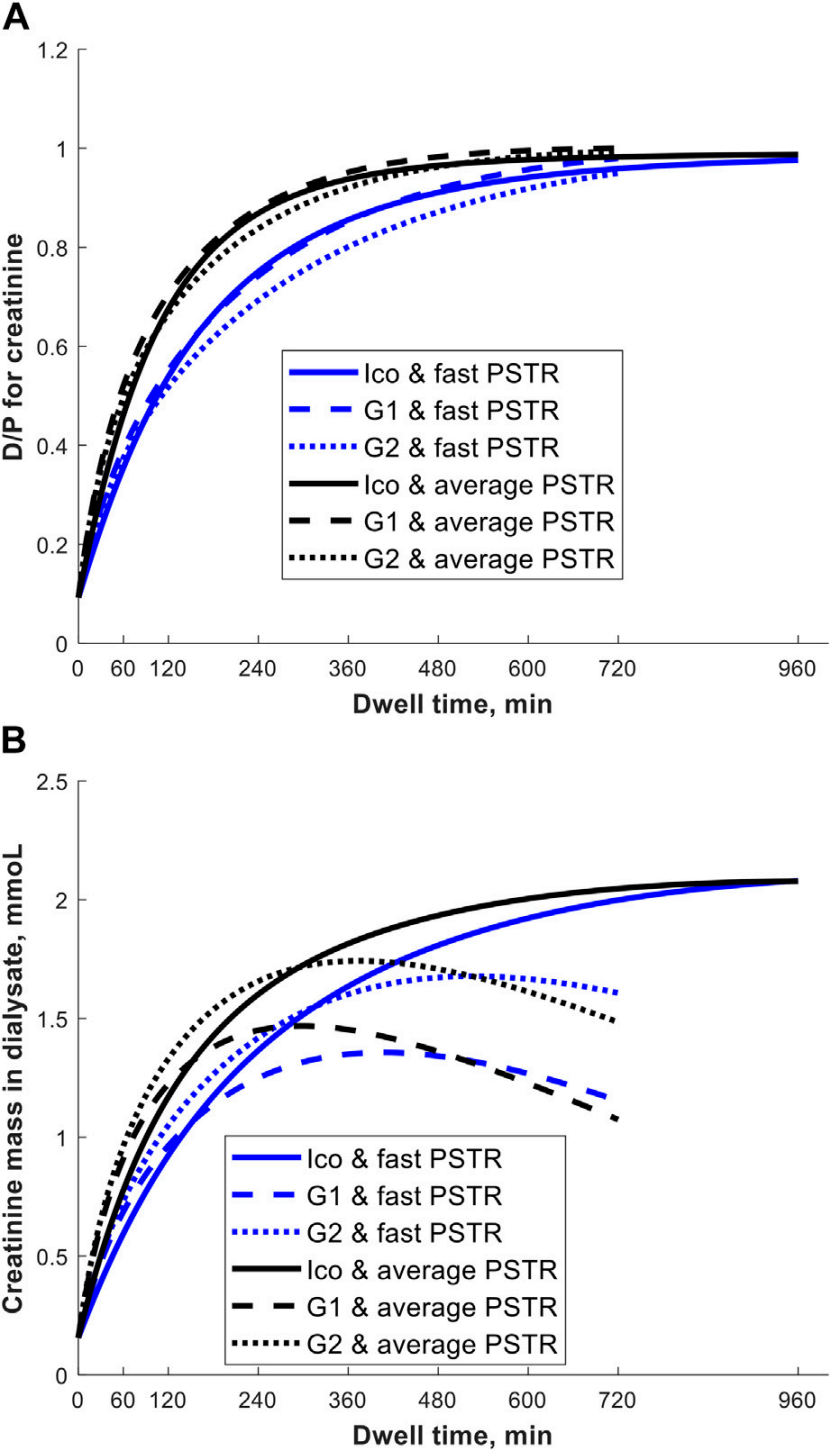
Creatinine dialysate-to-plasma concentration, D/P (panel A), and creatinine mass in dialysate (panel B) as a function of dwell time during the 12-h exchange with 1.36% (G1) or 2.27% (G2) glucose-based solution and 16-h exchange with 7.5% icodextrin-based solution (Ico, Extraneal^®^) in a patient with a fast and average PSTR.

**FIGURE 3:**
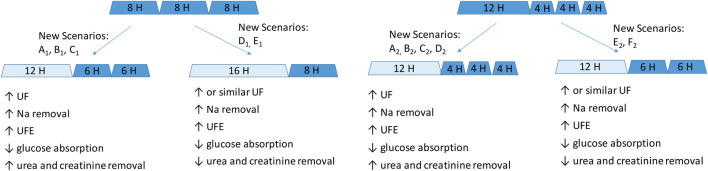
Schematic representation of the impact of replacing one or two glucose-based solutions with one long icodextrin exchange in patients with a fast and average PSTR. Glucose-based exchanges are denoted by dark blue, and icodextrin-based exchanges, by light blue. New scenarios refer to the detailed data presented in Tables 2–5.

Why could one exchange of 7.5% icodextrin replace two shorter exchanges of 1.36% or 2.27% glucose solutions? First, this prescription change results in adequate fluid removal. Numerical simulations in our study suggest higher efficiency of icodextrin in water removal, i.e., higher UFE, in the case of longer dwells with a duration of over 10 h, whereas glucose-based solutions are more beneficial in the case of shorter dwells (Figure 1). Unlike glucose-based solutions, icodextrin resulted only in minor differences between transport groups in terms of water and solute removal, with UFE remaining stable up to 16 h (Table 1). Second, the introduction of 7.5% icodextrin also brings relative sufficient solute removal. Although the equilibration rates (in terms of the dialysate to the plasma concentration rate) for urea and creatinine are similar for icodextrin and glucose solutions, the water transport influences the observed removal of urea and creatinine (Figure 2). In consequence, a decrease in ReU and ReCr with increased dwell duration is observed for glucose-based solutions, whereas a persistent increase occurs during icodextrin dwells. Furthermore, despite higher removal during shorter glucose dwells, more efficient removal of urea and creatinine is provided by icodextrin in the case of longer dwells (Figure 2; Table 1). Based on the latest ISPD guideline (Brown et al., 2020), we shall consider more aspects regarding dialysis adequacy, including patient reported outcomes, fluid removal, and nutrition, whereas the view that small-solute removal (urea, creatinine, etc.) is the only important index to consider when evaluating dialysis quality is no longer valid. However, we need further clinical studies to verify if the new prescription provides patient benefits. Obviously, the replacement of two glucose exchanges by a single exchange with icodextrin solution provides higher or similar water removal and higher sodium daily removal for patients from average and fast transfer groups. Third, the use of one 7.5% icodextrin solution instead of two glucose solutions also leads to lower glucose uptake, slower dissipation of the osmotic agent, and higher UF efficiency (Table 2). However, this change leads also to a slightly lower daily removal of urea and creatinine, irrespective of the transport type in the case of reference prescriptions with 3–4 daily glucose-based exchanges (Tables 2, 3). The direction of changes induced by the replacement of 1–2 glucose-based exchanges by one long dwell with 7.5% icodextrin is the same for all investigated reference prescriptions with 3–4 exchanges per day and patients with a fast and average PSTR, as summarized in Figure 3.

Recently reported findings showed that the method of steady-concentration PD performed using a device providing continuous glucose infusion to maintain a high intraperitoneal glucose concentration resulted in higher UF rates, more efficient use of glucose (increased ultrafiltration volume/gram glucose absorbed), and greater sodium removal than those when using standard 2.5% dextrose CAPD dwell (Heimburger et al., 2023). However, while the strategy of PD with a steady glucose concentration means a more efficient use of glucose as an osmotic agent, it is associated with increased exposure to glucose, which may have disadvantages because of harmful metabolic effects and negative effects on the peritoneum.

Our study has several limitations. First, the daily fluid and solute removal calculated in this study represents the removal related purely to peritoneal dialysis, without renal clearance taken into account, and we did not analyze the impact of dry/wet periods. Moreover, the presented simulations consider only the dwell time, whereas the impact of the infusion and drainage procedures on fluid and solute transport was not included in the present model. We also do not provide results of simulations with 3.86% glucose, a solution which is highly efficient in terms of fluid and solute removal in short dwells also in patients with a fast PSTR since this was outside the scope of our study. Strengths of the study include the use of detailed clinical data on fluid and solute transfer from studies in patients using glucose-based (Heimburger et al., 1992) and icodextrin-based (Olszowska et al., 2019) solutions that were incorporated in a modified three-pore model with icodextrin hydrolysis taken into account (Akonur et al., 2015; Stachowska-Pietka et al., 2023).

In summary, simulations of the peritoneal transfer of fluid and solutes in patients with a fast or average PSTR who used 3–4 glucose-based (1.36% and 2.27%) exchanges per day showed that one icodextrin solution could replace two glucose-based exchanges. Although the new prescriptions with icodextrin resulted in increased water and sodium removal independent of the PSTR, there was only a minor reduction in urea and creatinine removal in the case of the replacement of two glucose-based exchanges. Unlike glucose-based dwells for which the removal of fluid and solutes becomes negative after 4–6 h in patients with a fast PSTR, icodextrin was associated with only minor differences between PSTR groups in terms of water and solute removal, which increased on average steadily throughout the long dwell up to 16 h, with UFE remaining close to 10 mL/g. These results suggest that dialysis schedules using one 7.5% icodextrin (Extraneal) exchange instead of two exchanges of glucose-based solutions might be a strategic prescription to provide adequate dialysis and potential benefits to the patient quality of life with less exposure to glucose. Further clinical studies are needed to confirm these findings.

## Data Availability

The raw data supporting the conclusion of this article will be made available by the authors, without undue reservation.
