# Supplementary material for: Can one long peritoneal dwell with icodextrin replace two short dwells with glucose?
Source: Front Physiol. 2024 Jul 10;15:1339762. doi: 10.3389/fphys.2024.1339762 (PMC11266149; doi:10.3389/fphys.2024.1339762)
Supplement: Supplementary file 1 [file DataSheet1.docx]

Supplementary Material

**Supplementary Table S1.** The model parameters assumed for computer simulations.

| Parameter | Value |
| --- | --- |
| Small pore radius (r_S_), Å | 43 |
| Large pore radius (r_L_), Å | 250 |
| Fractional small pore UF coefficient (α_SP_) | 0.85 |
| Fractional large pore UF coefficient (α_LP_) | 0.08 |
| Fractional ultrasmall pore UF coefficient (α_UP_) | 0.07 |
| Hydraulic conductivity (L_p_S), mL/min/mmHg | 0.068^a^ |
| ‘Unrestricted’ pore area over diffusion distance (A_0_/Δx), cm | 22 960^b^ |
| Peritoneal absorption (L), mL/min | 0.9^c^ |
| Transperitoneal oncotic pressure gradient (ΔΠ), mmHg | 22 |
| Transperitoneal hydrostatic pressure gradient (ΔP), mmHg | 8 |
| Dialysis instilled volume, mL | 2000 |
| Peritoneal residual volume, mL | 203^d^ |
| PS for urea, mL/min | 19.76 |
| Serum sodium concentration, mmol/L | 138^d^ |
| Serum glucose concentration, mmoL/L | 9.8^d^ |
| Serum urea concentration, mmol/L | 18^d^ |
| Serum creatinine concentration, mmol/L | 0.77^d^ |
| Dialysate sodium concentration, mmol/L | 132 or 133^e^ |
| Dialysate urea concentration, mmol/L | 0 |
| Dialysate creatinine concentration, mmol/L | 0 |
| Degradation rates for icodextrin fractions, (1/min)/(U/mL):  Fraction 1  Fraction 2  Fraction 3  Fraction 4  Fraction 5  Fraction 6  Fraction 7 | 0.0322  0.0214  0.0021  0.0009  0.0191  0.1122  0.4148 |

^a^ UF coefficients (total and for each pore type) were adjusted from 0.074 mL/min/mmHg that corresponds to the 25 000 cm to the estimated A0/Δx=22 960 cm obtained for a typical patient with average PSTR based on clinical data from (7).
^b^ 22 960 cm for an average PSTR, and inflated by 60% to 36 736 cm for fast PSTR, as proposed by (9).
^c^ It is a mean value of peritoneal absorption calculated using volume marker absorption from the peritoneal cavity for clinical data from (7) and (8)
^d^ Values taken from (6, 8)
^e^ Concentration of sodium: 132 mmol/L – for glucose 2.27% and 1.36% solutions, and
133 mmol/L – for icodextrin solution

**Supplementary Figure S1.** Intraperitoneal volume and small solute (glucose, sodium, urea, creatinine) concentration in dialysate as a function of dwell time. Solid lines correspond to the numerical simulations of the three-pore model and blue dots with bars corresponds to the mean data from peritoneal dwell with glucose 2.27% (1).

**Supplementary Figure S2.** Mean measured values of intraperitoneal volumes and small solute concentrations in dialysate: glucose, urea, creatinine, and icodextrin low molecular weight (Ico1) oligomers (mean values from all patients ± SD, blue dots) and in plasma (mean values, red crosses) taken from (5, 7) and mean simulation profiles for the minimal model (black solid lines) during 16-hour peritoneal dialysis with 7.5% icodextrin.

**Supplementary Figure S3.** Mean measured values of icodextrin high molecular weight (HMW) metabolites concentrations (Fractions 2-7 denoted as Ico2-7, mean values from all patients ± SD, blue) taken from (5, 7) and mean simulation profiles for the minimal model (black solid lines) in dialysate during 16-hour peritoneal dialysis with 7.5% icodextrin.
